# Treatment of the Dental Pulp

**Published:** 1876-12

**Authors:** Lane Clark


### TREATMENT OF THE DENTAL PULP.

BY LANE CLARK, D. D, S., R. C. S.

The methods of treating the dental pulp under its various conditions of disease are many and varied, and though each of its kind has a certain success, yet withal failures are in certain cases frequent, and we are again compelled to "try fresh fields and pastures new." Knowing this, I venture to add my mite to the troubled science. It has for some time appeared to me a strange way of restoring so delicate an organism as the pulp to health by treating it with such gentle means as strong nitric acid, arsenic and a few more remedies running from one extreme to the other. My failures have been so frequent in all that I have tried, that I was induced to seek for a body which would place the pulp under the same conditions as those in which it lived and flourished; in fact, to try and replace the lost dentine. I have found such means in gelatinised phospho-carbonate of lime, which is capable of being rendered very plastic for a short time and hardening very quickly. In pulps which have been exposed for some time I have found it requisite to mix with the compound a little tannic acid, but even in those cases I finish at one visit. I take care to saturate the cavity at starting with Glycerine, so that the air may be kept from the pulp while freely exposing it; after thoroughly removing all the decomposed parts, I place the gelatinised lime over the pulp, and using pressure until a slight sense of pain is felt, I accelerate the hardening of the lime with absolute alcohol. So soon as it is hard, I then treat as an ordinary case and plug at once. My success has been so constant with it that I have now lost all my former fears of failure in conservative treatment of the dental pulp. It may be that the means employed are not new, but whether it be so or not, it has proved in my practice a means of saving many a tooth otherwise condemned by the forceps.—*Monthly Review of Dental Surgery.*

